# Academic Resilience and Engagement in High School Students: The Mediating Role of Perceived Teacher Emotional Support

**DOI:** 10.3390/ejihpe11020025

**Published:** 2021-03-31

**Authors:** Luciano Romano, Giacomo Angelini, Piermarco Consiglio, Caterina Fiorilli

**Affiliations:** Department of Human Sciences, University of Rome LUMSA, 00193 Rome, Italy; g.angelini@lumsa.it (G.A.); piermarco.consiglio@gmail.com (P.C.); fiorilli@lumsa.it (C.F.)

**Keywords:** academic resilience, perceived teacher support, school engagement, high school students

## Abstract

Academic resilience is the ability to overcome setbacks and chronic difficulties in the academic context. Previous studies have found that resilient students tend to be more engaged in school than their counterparts. Nevertheless, it seems worth deepening the role of contextual factors, such as teacher emotional support and how students perceive it, as it could contribute to foster the abovementioned relationship. The present study aimed to examine the links between academic resilience, perceived teacher emotional support, and school engagement. Moreover, the mediating role of perceived teacher emotional support was investigated. A sample of 205 Italian high school students (58.5% female), aged 14–19 years (M = 16.15, SD = 1.59), completed self-report questionnaires on academic resilience, perceived teacher emotional support, and school engagement. A structural equation model (SEM) was used to test the mediation hypothesis. The results showed that academic resilience was associated with perceived teacher emotional support, and both of them were related to school engagement. Furthermore, perceived teacher emotional support partially mediated the relationship between academic resilience and school engagement. Findings were discussed by underlining the importance of fostering personal and contextual resources in the school context to promote students’ well-being.

## 1. Introduction

In the last few decades, academic resilience has gained increasing attention in the school context due to its relation to positive achievement and school-related adjustment [1,2]. Resilient students, indeed, seem to be able to successfully overcome stressful school-related conditions, maintain optimal levels of motivation, and gain high performance despite the difficulties [3,4]. Previous studies have deepened the relationship between academic resilience and school engagement, showing that they are strictly and positively related (e.g., [5]). When dealing with school tasks, students who rely on personal resources, such as academic resilience, tend to drive all the efforts to achieve their goals, enhance their energy and dedication levels in daily activities and be more engaged than their peers [6,7,8]. Additionally, further studies have underlined that external resources play a synergetic effect with personal ones in affecting individuals’ engagement [9,10]. It seems that individuals with higher personal resources seek contextual resources to help them succeed. It influences the way they perceive the environment and the social support they receive [11]. In line with this evidence, a recent study has highlighted that external resources, such as perceived academic support, partially mediate the relationship between students’ personal resources and engagement [12]. More specifically, resilient students perceive and recruit teachers’ support to enhance their engagement. Despite the abovementioned evidence, it seems worth expanding the previous findings by deepening contextual resources’ role and focusing on emotional forms of perceived support, such as students’ perceived teachers’ emotional support. In detail, it refers to students’ perception of whether and how teachers show caring and attentive behaviors to their socioemotional needs [13]. Especially in the high school context, teachers’ emotional interactions are crucial for students’ well-being and engagement [14]. Therefore, the present study aimed to investigate the relationship between academic resilience and school engagement by analyzing the mediating role of perceived teacher emotional support. 

### 1.1. Academic Resilience and School Engagement

Resilience is widely conceptualized as the individual characteristic, capability, or process to positively adapt to challenges and overcome threatening events [15]. Similarly, academic resilience is viewed as the ability to successfully face setbacks and chronic difficulties in the academic context [3,4]. Several authors have considered academic resilience as a unique characteristic of students arising from specific and dramatic conditions, such as low socioeconomic backgrounds or extremely traumatic life events [16,17]. Despite this, further studies suggested that academic resilience is a relevant feature identified in all students who face severe adversities during their academic path [18,19]. Resilient students are the ones who reengage and do not give up when dealing with overwhelming academic tasks. This feature is predictive of several positive outcomes, such as enjoyment of school, class participation, and general self-esteem [3,20,21]. 

Further studies have shown that academically resilient students are more shielded from severe forms of maladjustment, report higher academic performance, and seem more engaged in the school context than their counterparts [22,23,24]. Although academic literature had somewhat reported contrasting results on the directionality of the relationship between resilience and engagement in the school context [2,25,26], a recent systematic review posited that school engagement should be considered a positive adaptation outcome of academic resilience [27]. School engagement is a multidimensional construct that includes cognitive, affective, and behavioral components [28,29] and encompasses three dimensions: vigor, dedication, and absorption [30,31]. While vigor reflects motivation and high levels of energy, dedication is related to schoolwork investment and a positive attitude towards studying. Finally, absorption regards being so concentrated in their work that students do not notice that time passes rapidly [28,30,32]. In line with the study of Tudor and colleagues [27], further studies highlighted that academic resilience and its components were predictive of higher school engagement levels [5,33,34]. Furthermore, the demand-resources model applied to the educational tracks posited that accounting for higher personal resources (i.e., academic resilience) might promote academic goals and school engagement [6,12,24,35]. Thus, assuming academic resilience as a personal resource and antecedent of school engagement, the current study aimed to deepen the association between these variables.

### 1.2. The Mediating Role of Perceived Teacher Emotional Support

Adolescents spend most of their daily time in the classroom, have to face new and complex school tasks, and deal with different kinds of pressure and expectations [36]. In light of the emotional burden related to overwhelming school demands and the abovementioned evidence, it is not surprising that they are significantly influenced by the emotional support received from significant adults in the school environment [14,37,38]. Teacher emotional support refers to a teacher’s ability to create positive relationships with their students and promote their autonomy and socioemotional functioning [14,39]. According to Pianta and Hamre [40], teacher emotional support embraces three dimensions: positive climate, teacher sensitivity, and regard for adolescent perspective. Positive climate refers to how a teacher is keen on creating positive interactions with their students. Teacher sensitivity pertains to whether and how a teacher can respond to students’ academic and emotional needs. Finally, regard for adolescent perspective concerns the degree to which a teacher encourages students’ autonomy, interaction with peers, and whether she/he is attentive to their social needs [14,41]. Students in classrooms with emotionally supportive teachers tend to report high performance and school adjustment [36,42,43]. Further studies also highlighted that students who perceived their teachers as emotionally supportive are better motivated and engaged in their studies than their counterparts (e.g., [44]). Besides, previous scholars have shown that resilient individuals, thanks to their high positive emotionality, can arouse warmth and positive emotion in close others, promoting an emotionally supportive environment (e.g., [45]). In line with these findings, several studies underlined that resilient students report higher levels of perceived social support and tend to perceive their learning environment more positively than their low-resilient peers [46,47,48,49]. Echoing these findings, further studies have shown that academically resilient students, compared to their peers, are more likely to perceive warmth support from their teachers, which is related to greater adjustment in the long term (e.g., [50]). Moreover, a recent study has highlighted that academic resilience and perceived teacher support were both significantly and positively related to students’ engagement [5]. Thus, it is possible that resilient students tend to perceive higher emotional support from their teachers, which could lead them to be more engaged in the school context. 

### 1.3. Aims and Hypotheses

The present study sought to examine the relationship between academic resilience and school engagement in a sample of high school students. Furthermore, the mediating role of perceived teacher emotional support was investigated. Thus, we hypothesized that academic resilience was positively related to perceived teacher emotional support and school engagement (Hypothesis 1); perceived teacher emotional support was positively associated with school engagement (Hypothesis 2). Finally, perceived teacher emotional support mediated the relationship between academic resilience and school engagement (Hypothesis 3).

## 2. Materials and Methods

### 2.1. Participants

A convenience sample of 205 Italian high school students (F = 58.5%), aged 14 to 19 years (M = 16.15, SD = 1.59), participated in the study. Students belonged to two different high schools in Central (85.4%) and Southern Italy (14.6%). Specifically, 55.6% attended a high school focusing on classical studies (e.g., Greek and Latin), while 44.4 % attended a high school focusing on social science and humanities. The initial sample was composed of 207 students, there were no missing data, but two students were excluded from the analyses due to outliers.

### 2.2. Instruments

Academic resilience. Academic resilience was measured by the academic resilience subscale of the Italian Questionnaire for Anxiety and Resilience (QAR; [51]). The subscale is composed of 7 items on a 5-point Likert scale (1 = “Not at all,” 5 = “Totally”); an example of an item is “I overcome the agitation and the tension, and I bounce back from the moments of difficulty in the study.” The scale was used in previous studies, showing good psychometric properties [24,41]. In the current study, Cronbach’s alpha was 0.72.

Perceived teacher emotional support. The Italian validated version of the Teacher Emotional Support Scale [14] was used to measure students’ perception of their teachers’ emotional support. The scale is composed of 15 items on a 5-point Likert scale (1 = “Not at all true,” 5 = “Very true”). It measures three distinct and related aspects of teacher emotional support: Positive Climate (e.g., “Our teachers want students in this class to respect each other’s ideas”); Teacher Sensitivity (e.g., “Our teachers consider students’ feelings”); and, Regard for Adolescent Perspective (e.g., “Our teachers encourage us to help other students with their works”). Cronbach’s alpha was 0.94 for the total score in the current study and between 0.81 and 0.90 for its subscales.

School engagement. The Italian validated version of the Utrecht Work Engagement Scale-Student version (UWES-S; [28]) was used to assess school engagement. The scale is composed of 17 items on a 7-point Likert scale (0 = “Never,” 6 = “Always”). It comprised three subscales, namely Vigor (e.g., “I feel full of energy when I am studying”), Dedication (e.g., “My studies inspire me”), and Absorption (e.g., “I get drawn into my studies”). Cronbach’s alpha was 0.95 for the total score and between 0.83 and 0.95 for its subscales in the current study.

### 2.3. Procedure

The current study was conducted in Italy in February 2020, adopting a cross-sectional descriptive design. The research protocol was approved by the school council and the school principal. Furthermore, students were informed of the possibility to take part in the study by internal school communication. Participation was voluntary. Only students who gave their written informed consent and only under-age students whose parents gave their written informed consent took part in the study. Students compiled a self-report questionnaire with a paper–pencil approach during regular school hours, and a member of the research team was present in classrooms in case of need. Students received all the necessary information to fill out the questionnaire, and anonymity and confidentiality standards were ensured for all the participants. The research protocol was in accordance with the Declaration of Helsinki of 1964 and its latest versions. The study procedures received approval from the Ethics Committee of Lumsa University of Rome, Italy.

### 2.4. Analysis Plan

To verify the variables’ adequate normality, preliminary descriptive statistics, such as skewness and kurtosis, were performed, using SPSS v. 21.0 (Statistical Product and Service Solutions, Chicago, IL, USA). As skewness and kurtosis were not >2, a normal distribution was assumed, and the maximum likelihood estimation method (ML) was adopted. Moreover, Pearson correlations were performed to verify the associations among the studied variables. Gender variable was treated as a dummy variable where 0 = Female and 1 = Male.

**Figure 1:**
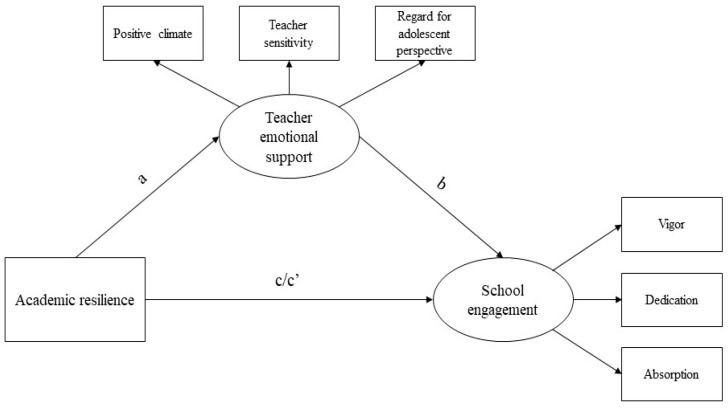
Conceptual mediation model to test Hypothesis 3 concerning teacher emotional support mediating role in the relationship between academic resilience and school engagement (N = 205). Paths from age and gender were excluded for presentation purposes.

Concerning the model specification, academic resilience was the predictor, while teacher emotional support was the mediator variable, and school engagement the outcome. For academic resilience, a composite score was used to obtain a global score, in line with previous studies [51,52]. Teacher emotional support and engagement were used as latent variables, with their respective three subscales’ composite scores as indicators. Gender and age were controlled for in the whole model. The hypothesized model is shown in Figure 1. Specifically, as depicted in Figure 1, (a) represents the effect of academic resilience on teacher emotional support, (b) represents the effect of teacher emotional support on school engagement, (c) represents the total effect of academic resilience on school engagement, and (c′) represents the direct effect of academic resilience on school engagement.

To test the hypothesized mediation model, a Structural Equation Model (SEM) with 5000 resamples of bootstrapped estimates with a 95% confidence interval (CI) was performed, using Mplus v. 8.3 (Muthén & Muthén, Los Angeles, CA, USA) and following Preacher and Hayes [53]. To verify the goodness of fit of the model, the following fit indices were used: Chi-squared test (*p*-value > 0.05 indicate a good fit), the comparative fit index (CFI), and the non-normed fit index (TLI) (values > 0.90 indicate a good fit; values > 0.95 indicate a very good fit), the root mean square error of approximation (RMSEA), and the standardized root mean square residual (SRMR) (values < 0.08 indicate a good fit, values < 0.05 indicate a very good fit).

## 3. Results

### 3.1. Descriptive Statistics and Correlations

Table 1 shows the means, standard deviations, minimum, maximum, skewness, kurtosis, and correlation matrix.

Results support Hypothesis 1 by showing that academic resilience is positively and significantly related to students’ perceived teacher emotional support (*p* < 0.01) and school engagement (*p* < 0.01). Therefore, the more students are resilient, the more they perceive their teachers’ emotional support and show higher engagement. Moreover, findings support Hypothesis 2, highlighting that students’ perceived teacher emotional support is significantly and positively related to school engagement (*p* < 0.01). Thus, students who perceive higher teachers’ emotional support also report higher levels of school engagement.

### 3.2. Mediation Analysis Results 

The structural equation model (SEM) performed to test the mediation of teacher emotional support in the relationship between academic resilience and school engagement shows a good fit to the data. Specifically, χ^2^ (16) = 32.689, *p* < 0.01; root mean square error of approximation (RMSEA) = 0.07; standardized root mean square residual (SRMR) = 0.02; comparative fit index (CFI) = 0.98; non-normed fit index (TLI) = 0.96.

**Figure 2:**
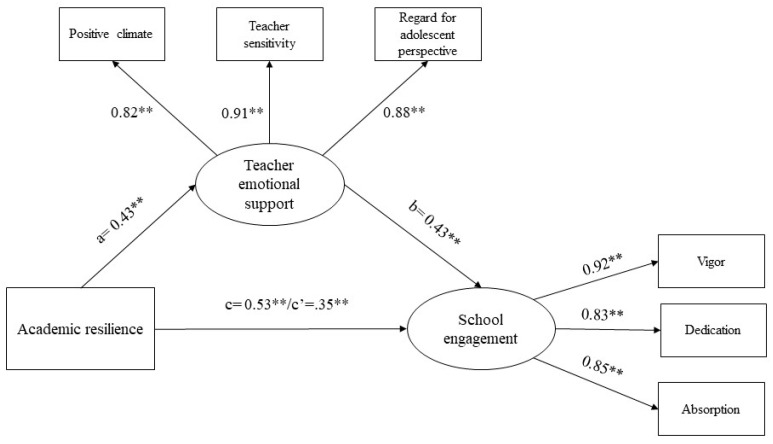
Structural mediation model between academic resilience, teacher emotional support, and school engagement (N = 205). The standardized effects are reported. Paths from age and gender were excluded for presentation purposes. ** *p* < 0.01.

In line with the mediation testing analysis, the hypothesized structural path (see Figure 1) was performed estimating the following effects: the effect of academic resilience on perceived teacher emotional support (a); the effect of perceived teacher emotional support on school engagement (b); and both the total (c) and the direct (c′) effect of academic resilience on school engagement. The age and gender variables have been controlled for in the whole model. Results of the structural mediation model are shown in Figure 2.

The direct paths show that academic resilience is significantly and positively related to school engagement (β = 0.35, *p* < 0.001, 95% CI: [0.25, 0.43]) and teacher emotional support (β = 0.43, *p* < 0.001, 95% CI: [0.32, 0.54]). Higher resilience in our students is associated with higher perceived teacher emotional support and engagement. Furthermore, teacher emotional support is significantly and positively related to school engagement (β = 0.43, *p* < 0.001, 95% CI: [0.33, 0.53]). Thus, higher perceived emotional support provided by teachers is linked to higher engagement levels in students. Age is significantly and negatively related to school engagement (β = −0.18, *p* < 0.001, 95% CI: [−0.25, −0.10]) and teacher emotional support (β = −0.44, *p* < 0.001, 95% CI: [−0.55, −0.34]), while gender is significantly related to teacher emotional support (β = 0.26, *p* < 0.001, 95% CI: [0.12, 0.39]). Specifically, younger students in our sample tend to perceive higher emotional support from their teachers and be more engaged than their older peers. Moreover, male students perceive higher teacher emotional support from their teachers than their female classmates. 

Furthermore, supporting Hypothesis 3, the path of academic resilience-school engagement is significantly mediated by teacher emotional support, accounting for 46% of the total variance. In detail, the analyses of the total (β = −0.53, *p* < 0.001, 95% CI: [0.44, 0.62]), direct (β = 0.35, *p* < 0.001, 95% CI: [0.25, 0.43]) and indirect effects (β = −0.18, *p* < 0.001, 95% CI: [0.11, 0.26]) of academic resilience on school engagement suggest that perceived teacher emotional support partially mediates the relationship between academic resilience and school engagement. Thus, a higher academic resilience is related to a higher perceived teacher emotional support, which leads to higher school engagement levels. 

The statistical significance of standardized direct, indirect, and total effects (and their relative bootstrap 95% CI) is summarized in Table 2.

## 4. Discussion

The present study sought to investigate the relationship among academic resilience, perceived teacher emotional support, and school engagement in a sample of high school students. Specifically, the main aim was to examine whether academic resilience was related to their school engagement levels via perceived teacher emotional support. Findings supported both the correlational and the mediational hypotheses. 

In detail, our results supported Hypothesis 1, showing positive correlations between academic resilience on the one hand and perceived teacher emotional support and school engagement on the other. Previous studies have shown that resilient individuals perceive higher social support than their counterparts [48]. Besides, scholars have previously highlighted the inter-relationship between academically resilient students and teachers who create a warm classroom community [54]. Similarly, further studies have found that students with resilient characteristics were more predisposed to school engagement [33]. Previous studies have underlined that commitment, one of the constitutive features of academic resilience [3], is strongly related to school engagement and overall school adjustment [55]. Thus, the current findings underlined that academically resilient students in our study were also more likely to perceive higher teacher emotional support and be more engaged in the school context.

Furthermore, our results supported Hypothesis 2 by showing that perceived teacher emotional support and engagement were positively associated. Students who perceived higher teacher emotional support tend to be more engaged in the school context, and this datum echoed previous findings (e.g., [44,56,57]). Specifically, further studies have underlined the association between teacher emotional support and student engagement [58,59]. Moreover, recent research has shown that the dimensions of perceived teacher emotional support and engagement were strictly related in high school students [14].

Besides, our findings supported Hypothesis 3, revealing that perceived teacher emotional support partially mediated the path of academic resilience-school engagement. Previous studies on teacher support’s mediating role toward school engagement reported inconsistent results (e.g., [60]). In contrast, further studies have found that resilience acted as a mediator in the relationship between teachers’ ability to create a good school climate and positive academic outcomes (e.g., [61]). Thus, and to the best of our knowledge, no previous studies have deepened whether and how students’ perception of contextual resources (i.e., teacher emotional support) could intervene in the link between academic resilience and school engagement. Our results showed that academically resilient students tend to be more engaged in school when facing school demands, thanks to a higher perception of their teachers’ emotional support. This datum could be better understood in light of previous findings. Specifically, a previous study highlighted that teacher support plays a pivotal role in resilient students’ engagement process [62]. More in-depth, the authors posited that resilient students tend to establish a reliable, supportive network in school and have trusting relationships with significant others in the school environment, as they believe teachers could foster their engagement and academic success [62].

Further studies underlined that academically resilient students are more engaged in school activities [63], showing more enjoyment at school, as well as positive interactions with teachers, and higher class participation than their low-resilient peers [3]. Moreover, previous scholars have also shown that resilient students adopted higher support-seeking strategies [64] and have a higher perception of support than other students [49]. Interestingly, a recent study highlighted that resilient students tend to adopt both problem-focused and emotion-focused coping strategies proactively when dealing with stressful situations [65]. Unlike their low-resilient peers, academically resilient students believe that venting their emotions could be part of the solution and not part of the issue when dealing with academic tasks. In other words, academically resilient students try to do the best possible to succeed and to engage in the school environment, exploiting the contextual and affective resources at their disposal at best, including their teachers’ emotional support [21]. Effectively, previous scholars highlighted that teachers’ interpersonal relationship is the key to reengage and bounce back from difficulties, contributing to students’ success [66,67,68].

The present findings shed light on the underlying processes that lead academically resilient students to be more engaged in the school context. Echoing previous findings [12,21,69,70]), our results underlined that personal resources (i.e., resilience) are determinant as much as contextual ones (i.e., teacher emotional support) in fostering students’ engagement. This evidence gives new insight in terms of practical interventions that could be employed in the school context. Firstly, our results underline the importance of promoting resilience in the school context to foster students’ engagement and prevent maladjustment. Several authors, indeed, have shown the effectiveness of resilience-based interventions to foster students’ well-being (e.g., [71,72]).

Moreover, our results give attention to effective classroom interactions, especially concerning teacher emotional support’s pivotal role as the current study’s core finding. In other words, the quality of the emotional closeness established with teachers could not be disregarded.

Finally, two interrelated issues come from our results. First, in our theoretical model, the student’s academic resilience plays a primary role. Effectively, how students face school-related events through their characteristics (i.e., resilience) can affect school careers (i.e., engagement). However, this finding should not distract the educators’ attention from the educational systems’ role. Academic resilience is due to several children’s life experiences where the relationships play a central role with adults (e.g., teachers). Second, and interrelated with the former point, teachers’ competencies to create and maintain a warm classroom climate are vital. From the professional development perspective, our findings strongly endorse that supporting students requires a teacher’s training. In this regard, policymakers and practitioners should promote interventions and training to boost teachers who may experience complex school-related events during their professional careers. It is well known that teachers’ mental health and their work-related well-being impact their students’ well-being at school.

There are several limitations to this study that are important to note. Among them, due to our study’s cross-sectional nature, it is impossible to draw causal relationships among the study variables. Moreover, considering the relatively balanced sample, it would be interesting in further studies to examine the moderating role of gender and other sociodemographic variables that have not been taken into account in the current study. In detail, concerning the role of gender, previous studies have underlined that Italian male adolescents tend to show higher resilient mechanisms than females [73]. Conversely, further studies have shown that, in stressful conditions, females are keener than boys in seeking support and report more positive connections with teachers [74]. Therefore, it might be worth deepening these aspects to expand our findings.

Furthermore, future studies could also test reversed models that have not been performed in the present study. Besides, it would be interesting to adopt a multi-informant design in future studies by collecting teachers’ reports, as we did not do so in the present study. It is possible that our sample teachers have a different perception of the emotional support provided to their students, and it would be interesting to analyze the potential agreement between the two informants.

## Figures and Tables

**Table 1:** Descriptive statistics and correlation matrix.

Variables	*M*	*SD*	Min	Max	Skewness	Kurtosis	2	3	4	5
1. Gender	-	-	-	-	-	-	-	0.181 **	−0.241 **	0.040
2. Age	16.15	0.11	14	19	0.27	−1.15		−0.057	0.344 **	0.136 *
3. AR	22.70	0.34	7	35	−0.06	0.45			0.491^**^	0.555 **
4. ES	44.99	0.95	15	75	−0.22	−0.20				0.540 **
5. UWES-S	61.17	1.62	17	102	0.25	−0.24				

*Note.* **. *p* < 0.01 (2-tails), * *p* < 0.05 (2-tails); AR = Academic resilience; ES = Perceived teacher emotional support; UWES-S = School engagement.

**Table 2:** Results of mediation with standardized effect and bootstrap 95% CI.

Effects	School Engagement [95% CI]
Total	0.53 *** [0.44, 0.62]
Indirect via teacher emotional support	0.18 *** [0.11, 0.26]
Direct	0.35 *** [0.25, 0.43]

*Note.* *** *p* < 0.001.
